# Deep transcranial magnetic stimulation add-on for the treatment of auditory hallucinations: a double-blind study

**DOI:** 10.1186/1744-859X-11-13

**Published:** 2012-05-06

**Authors:** Oded Rosenberg, Roman Gersner, Limor Dinur Klein, Moshe Kotler, Abraham Zangen, Pinhas Dannon

**Affiliations:** 1 Beer Ya'acov Mental Health Center, Beer Ya'acov, Israel; 2 Department of Neurology, Children's Hospital, Harvard Medical School, Boston, MA, USA; 3 Department of Life Science, Ben-Gurion University, Beer-Sheva, Israel

## Abstract

**Background:**

About 25% of schizophrenia patients with auditory hallucinations are refractory to pharmacotherapy and electroconvulsive therapy. We conducted a deep transcranial magnetic stimulation (TMS) pilot study in order to evaluate the potential clinical benefit of repeated left temporoparietal cortex stimulation in these patients. The results were encouraging, but a sham-controlled study was needed to rule out a placebo effect.

**Methods:**

A total of 18 schizophrenic patients with refractory auditory hallucinations were recruited, from Beer Yaakov MHC and other hospitals outpatient populations. Patients received 10 daily treatment sessions with low-frequency (1 Hz for 10 min) deep TMS applied over the left temporoparietal cortex, using the H1 coil at the intensity of 110% of the motor threshold. Procedure was either real or sham according to patient randomization. Patients were evaluated via the Auditory Hallucinations Rating Scale, Scale for the Assessment of Positive Symptoms-Negative Symptoms, Clinical Global Impressions, and Quality of Life Questionnaire.

**Results:**

In all, 10 patients completed the treatment (10 TMS sessions). Auditory hallucination scores of both groups improved; however, there was no statistical difference in any of the scales between the active and the sham treated groups.

**Conclusions:**

Low-frequency deep TMS to the left temporoparietal cortex using the protocol mentioned above has no statistically significant effect on auditory hallucinations or the other clinical scales measured in schizophrenic patients.

**Trial Registration:**

**Clinicaltrials.gov identifier: **NCT00564096.

## Introduction

Auditory hallucinations are reported by 50% to 70% of patients with schizophrenia, most of whom are successfully treated with antipsychotic medications. However, 25% to 30% of hallucinating schizophrenic patients are refractory to antipsychotic medications, and these patients suffer associated distress, functional disability, lack of behavioral control [1], and violent behavior [2]. Auditory hallucinations have also been known in up to 25% of the cases to contribute to serious suicide attempts [3]. Overactivation of the left temporoparietal cortex, which is critical to speech perception and is easily accessed through transcranial magnetic stimulation (TMS), has been implicated in the onset of auditory hallucinations [4].

The first report of repetitive TMS (rTMS) treatment for auditory hallucinations was described in 1999 by Hoffman [5]. Since then, several studies have used rTMS to treat auditory hallucinations in schizophrenic patients, targeting almost exclusively the left temporoparietal cortex, with mixed results [1,2,4,6,7]. The rationale for stimulating the left temporoparietal cortex with low frequency TMS is that imaging studies showed this area to be active during auditory hallucinations and low frequency TMS is thought to produce sustained reductions in neural excitability and brain activity in the stimulated region [2].

The H1 coil, used for deep TMS, has been demonstrated to be effective in the treatment of major depression [8-12]. A feasibility study of deep TMS as an add-on for treatment of negative symptoms and cognitive deficits in schizophrenia suggests that excitatory TMS applied to the prefrontal cortex might improve frontal lobe-related cognitive functions [13].

Deep TMS coils are designed to allow stimulation of deeper brain areas through the summation of separate fields projected into the skull from several points around its periphery [14]. The device is designed to minimize the accumulation of electrical charge on the surface of the brain, which can give rise to an electrostatic field that might reduce the magnitude of the induced electric field both at the surface and inside, reducing the depth of penetration of the induced electric field [15]. In an open label study [16] we previously examined the efficacy of deep TMS over the left temporoparietal cortex for the treatment of refractory auditory hallucinations in schizophrenic patients. Results were encouraging, but required a double-blind sham-controlled confirmation study.

## Materials and methods

### Participants

A total of 18 participants, 14 men and 4 women, were recruited for the study through outpatient clinics throughout Israel. All patients gave written informed consent to take part in the study, which was approved by the Beer-Yaakov ethics committee and Ministry of Health.

Inclusion criteria were: aged between 18 and 65, ability to give informed consent, meeting *Diagnostic and Statistical Manual of Mental Disorders, fourth edition text revision *(DSM-IV-TR) criteria for schizophrenia, experiencing auditory hallucinations at least five times per day, and taking a stable antipsychotic medication for at least 1 month prior to enrollment.

Eligible patients were randomized and assigned to either sham or real treatment. Patients as well as raters were blind to the type of treatment being given (real/sham). The stimulator was connected to a card reader. Magnetic cards coded for real/sham treatment were used. When a new patient was enrolled, a card was chosen randomly from the pack of cards.

### Real deep TMS group

The ages of participants in the group that received real deep TMS ranged between 19 and 63 years (average age was 40.8 ± 16.6). Eight were outpatients and one was an inpatient. The patients' hallucinations had persisted for an average of 4.4 (± 4.6) years prior to enrollment, despite adequate trials with an average of 4.1 (± 2) antipsychotic medications prior to study entry. The average age of disease onset was 26.4 (± 12.3) and the number of past hospitalizations averaged 3.7 (± 2.7).

### Sham deep TMS group

The ages of the sham group ranged between 22 and 63 years (average age was 38.4 ± 12.6). All were outpatients. Patients' hallucinations had persisted for an average of 9.2 (± 9.7) years, despite adequate trials with an average of 6.1 (± 2.4) antipsychotic medications prior to study entry. The average age of disease onset was 21.4 (± 7.7) and the number of past hospitalizations averaged 5.3 (± 6).

All participants were on antipsychotic medication during the study, with medication dosage kept stable throughout the study. Demographic data for all patients is presented in Tables 1 and 2. No significant differences were observed between the groups at baseline.

**Table 1:** Demographic table of participants in Sham arm.

Patient	Age	Sex	Diagnosis	Education (Years)	Status	Number of antipsychotic medications to which Auditory hallucinations were resistant	Time elapsed since present episode of Auditory hallucinations started (Years)	Number of past hospitalizations	Age of Disease onset
2-218	63	male	Schizophrenia	8	Ambulatory patient	10	10	20	18

4-218	43	male	Schizophrenia	12	Ambulatory patient	8	10	7	33

218-6	47	female	Schizophrenia	8	Ambulatory patient	6	32	2	15

218-7	46	male	Schizophrenia	8	Ambulatory patient	8	2	6	35

218-9	33	male	Schizophrenia	12	Ambulatory patient	4	1	3	19

218-10	32	male	Schizophrenia	9	Ambulatory patient	5	0.1	3	15

218-14	26	male	Schizophrenia	12	Ambulatory patient	2	6	0	20

218-15	34	male	Schizophrenia	14	Ambulatory patient	5	8	6	24

218-16	22	male	Schizophrenia	7	Ambulatory patient	7	14	1	14

**Table 2:** Demographic table of participants in real arm.

Patient	Age	Sex	Diagnosis	Education (Years)	Status	Number of antipsychotic medications to which Auditory hallucinations were resistant	Time elapsed since present episode of Auditory hallucinations started (Years)	Number of past hospitalizations	Age of Disease onset
218-2	62	female	Schizophrenia	13	Hospitalized	6	9	3	53

218-11	19	male	Schizophrenia	10	Ambulatory patient	1	0.5	1	18

218-12	20	male	Schizophrenia	12	Ambulatory patient	3	0.5	2	19

218-17	35	male	Schizophrenia	12	Ambulatory patient	2	8	1	27

218-18	38	male	Schizophrenia	12	Ambulatory patient	4	12	3	18

218-19	48	female	Schizophrenia	18	Ambulatory patient	6	No Data	5	37

218-21	42	male	Schizophrenia	8	Ambulatory patient	3	2	4	18

218-22	63	male	Schizophrenia	12	Ambulatory patient	7	0.3	5	32

218-23	26	male	Schizophrenia	10	Ambulatory patient	5	3	10	16

### Deep TMS procedure

We performed the treatments with a Brainsway H1 coil (Brainsway, Jerusalem, Israel), which has been tested in a safety study with healthy volunteers [17], and in a clinical study for the treatment of major depression [10]. The H1 coil's detailed configuration and electric field distribution maps have been described [18]. Stimulation was applied using Brainsway's H1 coil connected to a Magstim Rapid^2 ^stimulator (Magstim company limited, Carmarthenshire, Wales, United Kingdom). The resting motor threshold for each participant was obtained by stimulation of the left motor cortex, and defined as the minimum stimulator output intensity which caused a motor response, that is, twitching of the contralateral abductor policis brevis (APB) muscle in the hand.

The coil was then moved 4.5 cm posteriorly and 6.5 cm laterally towards the left shoulder of the patient. In this position, the maximal electric field produced by the coil is concentrated at the left temporoparietal cortex [16].

Patients were treated with deep H-coil TMS applied to the left temporoparietal cortex for 10 days (one session per day), with each session lasting 10 minutes. Treatment frequency was 1 Hz and the treatment intensity applied was 110% of the motor threshold.

### Sham deep TMS

Placebo stimulation was performed with a sham coil placed in the same helmet encasing the active TMS coil. An electronic system controlled which of the two coils was connected to the stimulator in a certain session. This operation was carried out by a magnetic card specific to each patient so that both the patient and the operator remained blind to the operation mode. The sham coil produces a similar acoustic artifact and scalp sensation as the active coil, and can also mimic the facial muscle activation induced by the active coil. However the sham coil induces an electric field of less than 30% of the size of the field induced by the real coil inside the brain itself, due to a very rapid reduction of the field as a function of distance insured by the non-tangential orientation of the sham coil relative to the scalp and by elements producing significant field cancellation.

### Patient assessment

#### Screening

Diagnoses were performed by trained psychiatrists using a semistructured clinical interview based on DSM-IV-TR criteria (Structured Clinical Interview for DSM (SCID), version 2), during which patients' main demographic and clinical characteristics were collected.

#### Efficacy

Each patient was evaluated within 24 h before the first TMS session, and within 24 h after the last TMS session, using the Auditory Hallucinations Rating Scale (AHRS) [2], the Scale for the Assessment of Positive Symptoms (SAPS), the Scale for the Assessment of Negative Symptoms (SANS), the Clinical Global Impressions scale (CGI), and the Quality of Life Enjoyment and Satisfaction Questionnaire (Q-LES-Q).

#### Safety

Adverse events (AEs) were evaluated during each study visit through communication with the patients.

### Statistical analysis

Results are presented as means ± SD. Differences between real and sham stimulation before and after treatment were determined by repeated-measures analysis of variance (ANOVA). Significant effects were found by Fisher's least significant difference post hoc test. All analysis was performed with Statistica 8.0. A *P *value of < 0.05 was considered statistically significant.

## Results

### Completer characteristics

Out of 18 patients, 10 (5 from each group) completed the study. Non-significant differences between the sham group to the real group in their clinical backgrounds were found, as outlined below.

In the sham group hallucinations had persisted for an average of 10 (± 12.8) years as opposed to 6.2 (± 4.9) in the real group. Auditory hallucinations of patients in the sham group were resistant to an average of 5.8 (± 2.5) antipsychotic medications compared to 4.4 (± 2) in the real group. In the sham group the average age of disease onset was 23.6 (± 9.7) compared to 29.6 (± 14.4) in the real group. The average number of past hospitalizations was 3.6 (± 2.8) in the sham group compared to 3.2 (± 1.4) in the real group.

### Detailed dropout reasons

The dropout rate was 44% in both the real and sham groups. In the real deep TMS group, four patients out of nine dropped out: one patient after four sessions because of a delusional thought (saying that the magnetic coil was 'pulling out his brain'), a second patient dropped out after two sessions because he was 'unable to tolerate the treatment', and a third patient dropped out after six sessions due to worsening psychotic symptoms (the patient became afraid of the stimulator). The participation of the fourth patient in the study was halted after nine sessions because of psychotic exacerbation. Of the five patients in the real deep TMS group that finished treatment, one did not complete the Q-LES-Q.

In the sham deep TMS group, four patients out of nine dropped out: one patient dropped out after four sessions after developing obsessive thoughts towards his treating psychiatrist as well as the desire to cut his wrists, saying he wanted to be admitted to the hospital and stop participating in the study. Another patient dropped out after two sessions claiming he could not stand the hitting of the coil against his scalp. A third patient dropped out after two sessions, as he had delusions that the coil was disfiguring his head. The fourth patient was reluctant to continue his participation after the fourth session (without any reasonable or psychotic explanation).

Patients that dropped out of either group were excluded from analysis.

### Efficacy measurements

**Figure 1:**
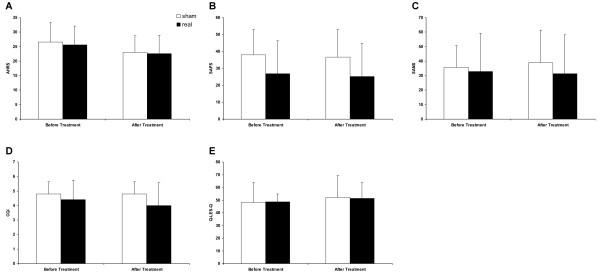
**Clinical measurements**. Data are presented as mean ± SD of Auditory Hallucinations Rating Scale (AHRS) **(A)**, Scale for the Assessment of Positive Symptoms (SAPS) **(B)**, Scale for the Assessment of Negative Symptoms (SANS) **(C)**, Clinical Global Impressions (CGI) scale **(D)**, and Quality of Life Enjoyment and Satisfaction Questionnaire (Q-LES-Q) **(E)**.

Statistics included all study completers (10 patients). The AHRS score post treatment was compared to the score prior to treatment. Minor decreases in auditory hallucinations were observed in both groups. AHRS scores were reduced from 25.6 ± 6.5 to 22.6 ± 6.2 in the active treatment group, and from 26.6 ± 6.5 to 23 ± 5.8 in the sham treatment group (Figure 1A). Repeated-measures ANOVA of AHRS with treatment as the between-subjects factor and time as the within-subjects factor revealed a significant main effect of time (F(1,8) = 5.54, *P *= 0.046) and no significant effects of group or interactions between the factors. Post hoc analysis did not show significant differences between the groups and indicated a non-significant tendency to decrease AHRS after either real or sham stimulation.

SAPS, SANS, CGI and Q-LES-Q scores post treatment were compared to the score prior to treatment. No difference was observed in either group for all measures (Figure 1B-E). Repeated-measures ANOVA with treatment as the between-subjects factor and time as the within-subjects factor did not reveal significant main effects of time, group, or time × group interaction.

### Adverse events

One patient suffered from mild and self-limiting headaches after the first two treatments. She did not use analgesics. Apart from that no side effects were observed.

## Discussion

We have tested deep rTMS treatment as an add-on to medication for refractory auditory hallucinations. The main finding of this study is that low-frequency deep transcranial magnetic stimulation to the left temporoparietal cortex given in the protocol mentioned above had no statistically significant effect on auditory hallucinations.

These findings are in agreement with Loo *et al.*'s findings [19], which suggested based on examinations of individually-controlled trials that a substantial proportion of rTMS studies of the treatment of auditory hallucinations did not find rTMS to be superior to sham stimulations. Our findings also correlate with a double-blind, randomized, sham-controlled study of 62 patients completed in March 2011 [20], which concluded that low-frequency rTMS administered to the left temporoparietal cortex (shown to be the site of maximal hallucinatory activation) is not more effective for medication-resistant auditory hallucinations than sham treatment. Tranulis *et al. *[21] conducted a meta-analytic study of 10 sham-controlled studies of low-frequency rTMS over the left temporoparietal cortex and found a medium statistical effect size. They reached the conclusion that rTMS is an effective tool as a complementary treatment for auditory hallucinations. On account of this contradictory data, the efficacy of low frequency rTMS over the left temporoparietal region is a matter that deserves further investigation.

It is important to note that the distribution of the H1 coil's magnetic field is both deeper and wider than the traditional figure-eight coil used in previous studies [22]. The H coils induce an effective field at a depth of approximately 3 cm below the skull, compared to less than 1.5 cm for the standard TMS figure-eight coil [22]. We would expect the H coil used in this study to be more effective than a figure-eight coil in treating auditory hallucinations due to its deeper penetration into the brain and because subcortical structures (the thalamus, for example) may be involved in the generation of auditory hallucinations [23]. However, we failed to achieve statistically or clinically meaningful improvements. One possible theoretical explanation may be that low frequency stimulation of subcortical structures counteracts effective stimulation of cortical zones (for example, the temporoparietal region).

The results of this study contradict our original open study [16] using the same deep TMS coil applied over the same location and using similar treatment parameters. This contradiction may stem from the fact that the placebo effect probably caused the positive results in the open study [16]. The placebo effect was weakened in this study because patients were oblivious to the kind of treatment (real/sham) they were given.

There are a few possible explanations for the inefficacy of the TMS presented in this study. The left temporoparietal cortex may not be the origin of auditory hallucinations in patients with schizophrenia, a possibility that may be supported by the fact that functional imaging studies showed increased activation of the right brain area in some patients suffering from auditory hallucinations. Other reasons may be inadequate/insufficient treatment parameters (including frequency, session duration, number of sessions, total pulses, or stimulation pattern).

The major limitation of our study is the small sample size. The negative result of this study may be false on account of the small sample size.

## Conclusions

Our study suggests that 10 sessions of low frequency deep TMS to the left temporoparietal cortex, given as add-on treatment to medication for refractory auditory hallucinations, is ineffective in ameliorating auditory hallucinations in schizophrenic patients. Larger trials are needed to establish this conclusion, since the validity of the result is weakened by our small sample size.

## Competing interests

PD and OR received an unrestricted educational grant for deep TMS treatment research from the Brainsway Company. RG is a scientific consultant of the Brainsway Company. AZ serves as a research consultant and has financial interest in the Brainsway Company. MK has no conflict of interest. LDK has no conflict of interest.

## Authors' contributions

OR participated in deep transcranial magnetic stimulation treatments described in the text, participated in writing the basic draft of the paper and rewriting the text according to coauthors suggestions, participated in writing the Discussion and Conclusions, and participated in clinical evaluations. RG participated by making extensive suggestions, advised on the Background, Methods, Discussion and Conclusions and conducted statistical analyses. LDK conducted evaluations and participated in writing the manuscript. MK participated in final approval of the manuscript. AZ participated by making extensive suggestions, advised on the Background, Methods, Discussion and Conclusions, and guided the paper scientifically. PD participated by contributing remarks and suggestions to the text, including the Discussion and Conclusions, supervised the DTMS sessions closely and conducted part of the deep transcranial magnetic stimulation treatments. All authors read and approved the final manuscript.
